# Embryonic Lethality of Mitochondrial Pyruvate Carrier 1 Deficient Mouse Can Be Rescued by a Ketogenic Diet

**DOI:** 10.1371/journal.pgen.1006056

**Published:** 2016-05-13

**Authors:** Benoît Vanderperre, Sébastien Herzig, Petra Krznar, Manuel Hörl, Zeinab Ammar, Sylvie Montessuit, Sandra Pierredon, Nicola Zamboni, Jean-Claude Martinou

**Affiliations:** 1 Department of Cell Biology, University of Geneva, Geneva, Switzerland; 2 Institute of Molecular Systems Biology, ETH Zürich, Zürich, Switzerland; Max Planck Institute for Biology of Ageing, GERMANY

## Abstract

Mitochondrial import of pyruvate by the mitochondrial pyruvate carrier (MPC) is a central step which links cytosolic and mitochondrial intermediary metabolism. To investigate the role of the MPC in mammalian physiology and development, we generated a mouse strain with complete loss of MPC1 expression. This resulted in embryonic lethality at around E13.5. Mouse embryonic fibroblasts (MEFs) derived from mutant mice displayed defective pyruvate-driven respiration as well as perturbed metabolic profiles, and both defects could be restored by reexpression of MPC1. Labeling experiments using ^13^C-labeled glucose and glutamine demonstrated that MPC deficiency causes increased glutaminolysis and reduced contribution of glucose-derived pyruvate to the TCA cycle. Morphological defects were observed in mutant embryonic brains, together with major alterations of their metabolome including lactic acidosis, diminished TCA cycle intermediates, energy deficit and a perturbed balance of neurotransmitters. Strikingly, these changes were reversed when the pregnant dams were fed a ketogenic diet, which provides acetyl-CoA directly to the TCA cycle and bypasses the need for a functional MPC. This allowed the normal gestation and development of MPC deficient pups, even though they all died within a few minutes post-delivery. This study establishes the MPC as a key player in regulating the metabolic state necessary for embryonic development, neurotransmitter balance and post-natal survival.

## Author Summary

The tight control of cellular metabolism and energy production plays a crucial role during embryonic development, cancer and neurodegenerative disorders. We show that mitochondrial pyruvate carrier deficiency in mice causes metabolic alterations that result in lactic acidosis, neurotransmitter imbalance, energy deficit, brain damage and embryonic lethality. Feeding the pregnant dams a ketogenic diet allowed the survival of affected embryos until birth. Our results demonstrate the importance of the mitochondrial pyruvate carrier in maintaining the metabolic program necessary to sustain normal mammalian development.

## Introduction

Pyruvate is a pivotal component in intermediary metabolism, lying at the crossroads between cytosolic and mitochondrial metabolism. The main intracellular source of pyruvate is glycolysis in the cytosol, which generates two molecules of pyruvate per molecule of glucose. Glycolysis-derived pyruvate then follows one of two major routes for energy production: conversion into lactate by lactate dehydrogenase (LDH) in a reaction that replenishes the cytosolic NAD+ cofactor pool, allowing maintenance of the glycolytic flux; or cytosolic pyruvate can enter the mitochondria to be oxidized to acetyl-CoA by the pyruvate dehydrogenase complex (PDH), fueling the TCA cycle and oxidative phosphorylation (OXPHOS). Alternatively, mitochondrial pyruvate can be used in an anaplerotic pathway through conversion to oxaloacetate by pyruvate carboxylase. In most differentiated cells, decarboxylation of pyruvate by PDH is used in order to meet the high energetic demands associated with specialized cellular processes such as the transmission of neuronal signals or muscle contraction [1]. In contrast, the strong anabolic requirements of proliferating cells are better met by high glycolytic rates, since several intermediates in this pathway serve as precursors for biomass production, including nucleotides and proteins synthesis [1,2]. This marked reliance on high glycolytic flux is a hallmark of highly proliferating cells, including many cancer cells in which a shift from oxidative phosphorylation to aerobic glycolysis (the Warburg effect) is frequently observed [2]. During embryogenesis, dynamic regulation of metabolic substrate utilization takes place, in which glucose utilization increases during the early, highly proliferative stages of development reaching a peak after embryo implantation. Vascularization and increased oxygen supply then trigger oxidative metabolism [1] allowing differentiation into different tissues [3,4]. Accordingly, mutations in glycolytic genes impair early post-implantation embryonic viability, while alteration in oxidative processes such as PDH activity often results in embryonic lethality at later stages (~E9-E11), when mitochondrial metabolism becomes crucial [1]. This versatility of metabolic pathways therefore allows the developing embryo to adapt its metabolism to meet the energetic and anabolic requirements of the diverse cellular programs.

In order to fuel the TCA cycle and drive oxidative phosphorylation, glucose-derived pyruvate must enter the mitochondrial matrix. To do so, it is believed to diffuse non-specifically through the outer mitochondrial membrane via porins [5], before being taken up by a specific carrier to cross the impermeable inner mitochondrial membrane. The existence of a specific transporter has been postulated since the 1970s [6], and its biochemical properties have been extensively studied, including its specific inhibition by chemical compounds [7,8]. However, the molecular and genetic identity of the mitochondrial pyruvate carrier (MPC) were revealed only recently by us and by others [9,10]. In mammals, the MPC is believed to be composed of two obligatory, interdependent subunits, MPC1 and MPC2, which form a multimeric complex of so far unknown stoichiometry [10] to mediate pyruvate transport across the inner mitochondrial membrane. These findings have led to renewed interest in the study of the physiological and pathological importance of the MPC, since they have allowed the development of genetic and biochemical strategies to investigate and experimentally modulate its expression, regulation and activity.

Recent studies in both yeast and mammalian cells have revealed a close and reciprocal relationship between MPC expression and activity and changes in cellular metabolic programs. When grown under fermentative conditions, yeast cells express a carrier composed of MPC1 and MPC2 subunits. In contrast, when grown under oxidative conditions they express MPC1 and MPC3 subunits, which results in the formation of a carrier with a higher efficiency for pyruvate import [11]. In turn, the importance of the MPC regulation as a driver of changes in cellular and whole organism metabolism is also beginning to be explored. In cell culture models, several recent studies have reported that pharmacological or genetic inhibition of the MPC resulted in a decreased contribution of glycolysis-derived pyruvate to the TCA cycle. Instead of leading to decreased TCA cycling, oxygen consumption and cell growth, the oxidative TCA cycle flux was maintained through an increase in glutamine-mediated anaplerosis and fatty acid oxidation. In addition, to compensate for the reduced pyruvate import, some pyruvate was found to be synthesized *in situ*, in the mitochondrial matrix, by mitochondrial malic enzymes [12,13]. Finally, in several cancers, a decrease in MPC activity has been observed [14–16], which correlates with poor prognosis in multiple colon cancers [16].

Several mouse models of MPC deficiency have now been published (*MPC2* hypomorphic allele [17], liver-specific knock-outs of *MPC1* or *MPC2* [18,19], acute MPC inhibition by UK5099 [20]), which showed defects in glucose-stimulated insulin secretion or gluconeogenesis, thus demonstrating a role for the MPC in regulating whole-body glucose homeostasis. However, ubiquitous disruption of MPC2 expression in mice results in embryonic lethality, and this has not been further investigated [17].

In this study, we have generated a whole-body knock-out of the *MPC1* gene and we have studied the impact of ubiquitous loss of MPC activity on mouse embryogenesis. We show that the loss of MPC1 protein results in embryonic lethality at E12-E14, and perturbations of respiratory and metabolic profiles in mouse embryonic fibroblasts (MEFs) derived from mutant embryos. These changes were reversed by re-expression of a functional MPC1 gene. In addition, mutant embryos presented lesions in the pons region of the brain stem, and the metabolome of the telencephalic brain showed significant anomalies, including lactate accumulation and an imbalance in the levels of several neurotransmitters. Interestingly, both lethality and the brain metabolism defects were prevented when the pregnant dam was maintained on a ketogenic diet. Overall, our results demonstrate that the MPC is required for normal mouse embryogenesis and brain development, but that the effects of its absence can be compensated, at least until birth, by a ketogenic diet.

## Results

### The *MPC1* gene trap allele efficiently silences MPC expression

**Fig 1:**
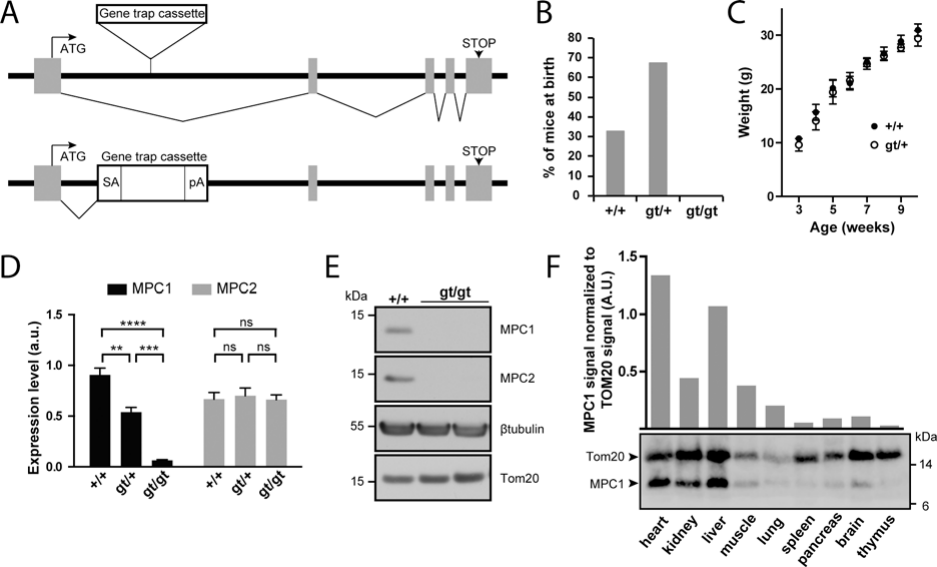
Disruption of the *MPC1* gene in mice. **(A)** A gene trap cassette inserted in the first intron of the *MPC1* gene (*MPC1*^*gt*^ allele) disrupts synthesis of full-length mRNA, abolishing the expression of MPC1 protein. SA, splice acceptor; pA, polyadenylation signal. **(B)** Genotype distribution of pups derived from heterozygous *MPC1*^*gt/+*^ breeding. **(C)** Weight gain profiles of *MPC1*^*+/+*^ and *MPC1*^*gt/+*^ mice from post-natal week 3 to week 10. **(D)** MPC1 (black bars) and MPC2 (grey bars) mRNA expression level (normalized to 28S rRNA) was evaluated by RT-qPCR in E13.5 embryos of the indicated genotypes. **p<0.01; ***p<0.001; ****p<0.0001 (two-way ANOVA). **(E)** MPC1 and MPC2 protein expression level was analysed by Western blot in *MPC1*^*+/+*^ and *MPC1*^*gt/gt*^ E13.5 embryos. β-tubulin and Tom20 were used as loading controls. **(F)** MPC1 protein expression in different tissues was analyzed by Western blot. The mitochondrial content of each of the tissues analyzed was evaluated using an antibody against the mitochondrial outer membrane protein Tom20.

To investigate the physiological importance of the MPC in vertebrates, we generated MPC1 deficient mice from an ESC clone harboring a gene trap cassette in the first intron of the *MPC1* gene (*MPC1*^*gt*^; **Fig 1A**). This cassette contains a splice acceptor at the 5’ end followed by transcription termination signals, thus disrupting MPC1 mRNA. After breeding *MPC1*^*gt/+*^ mice together, the resulting genotypes of the newborns diverged markedly from expected Mendelian ratios, with no homozygous *MPC1*^*gt/gt*^ pups being recovered (**Fig 1B**) suggesting that loss of MPC1 resulted in embryonic lethality as further described below. In contrast, heterozygous *MPC1*^*gt/+*^ mice appeared outwardly normal, showed no growth defect (**Fig 1C**) and were fully viable and fertile.

The efficiency of our gene disruption strategy was assessed by measuring MPC1 mRNA levels in embryos at E13.5. In *MPC1*^*gt/+*^ embryos, RT-qPCR showed that MPC1 mRNA levels decreased to about 50% compared to *MPC1*^*+/+*^ (WT), while in *MPC1*^*gt/gt*^ embryos, MPC1 mRNA was drastically reduced to about 5% of the WT level (**Fig 1D**). Nevertheless, this indicates that the gene trap allele is not 100% efficient and that at low frequency, splicing events may bypass the splice acceptor site of the cassette thus producing the full length MPC1 mRNA. However, we did not detect MPC1 protein by Western blot in E13.5 embryos (**Fig 1E**), showing that the amount of MPC1 protein in the *MPC1*^*gt/gt*^ embryos is, if present, very low. MPC2 mRNA levels remained unchanged in *MPC1*^*gt/gt*^ and *MPC1*^*gt/+*^ compared to WT (**Fig 1D**).

In WT adult mice, Western blot analysis showed that the level of MPC1 protein expression, after normalization to the mitochondrial marker Tom20, varies considerably in different tissues, with the highest expression levels in the heart and liver (**Fig 1F**). Nevertheless, it is widely expressed throughout the organism, and thus its absence could potentially affect the physiology of many vital organs.

### MPC1-deficient MEFs show impaired pyruvate-driven respiration

**Fig 2:**
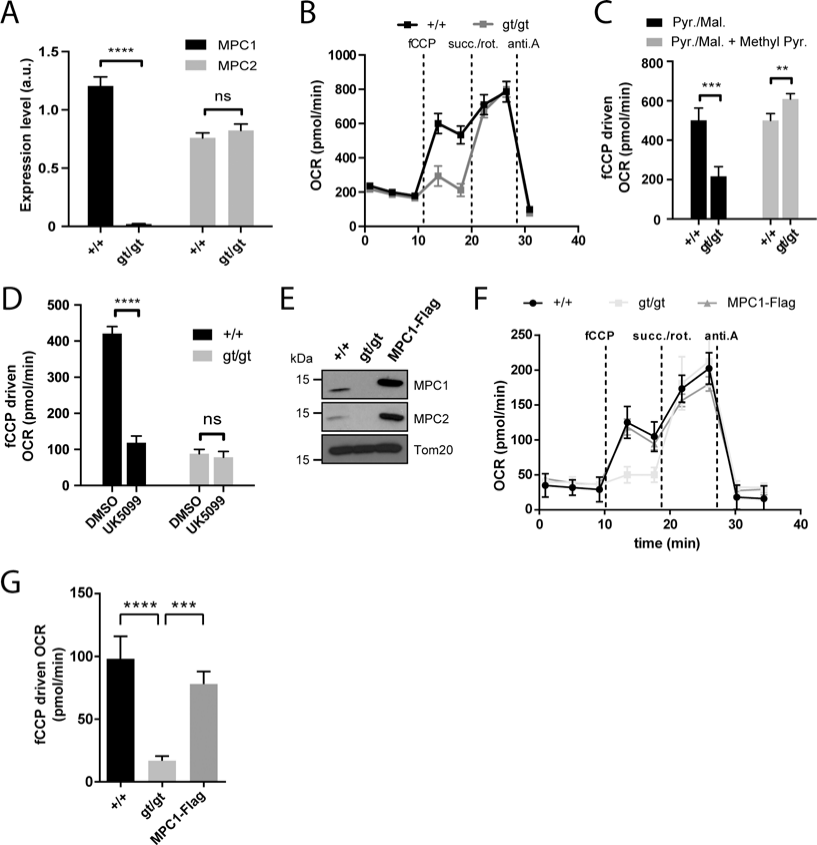
MPC1 is necessary for pyruvate-driven respiration. **(A)** MPC1 (black bars) and MPC2 (grey bars) mRNA expression level (normalized to 28S rRNA) were evaluated by RT-qPCR in *MPC1*^*+/+*^ and *MPC1*^*gt/gt*^ MEFs. ****p<0.0001 (two-way ANOVA). **(B)** Representative profiles of oxygen consumption rates (OCR) of XF PMP permeabilized primary MEFs with pyruvate/malate provided as carbon sources. fCCP: carbonyl cyanide-4-(trifluoromethoxy)phenylhydrazone; succ.: succinate; rot.: rotenone; anti.A: antimycin A. **(C,D)** fCCP-driven OCR of *MPC1*^*+/+*^ and *MPC1*^*gt/gt*^ permeabilized primary MEFs in the presence of pyruvate/malate (Pyr./Mal.) as carbon sources, and supplemented as indicated with membrane permeable methyl pyruvate (Methyl Pyr.)**(C)** or with the MPC inhibitor UK5099 or vehicle alone (DMSO)**(D)**. ** p<0.01; ***p<0.001; ****p<0.0001 (two-way ANOVA). **(E)** Western blot analysis of *MPC1*^*+/+*^, *MPC1*^*gt/gt*^ and *MPC1*^*gt/gt*^ MEFs stably expressing MPC1-Flag, using antibodies directed against MPC1, MPC2 and Tom20. **(F)** Representative OCR profiles of immortalized, permeabilized MEFs (*MPC1*^*+/+*^, *MPC1*^*gt/gt*^ and MPC1-Flag rescue cells) with pyruvate/malate provided as carbon sources. **(G)** fCCP-driven OCR from **(F)**; ***p<0.001 ****p<0.0001 (one-way ANOVA).

To assess the effect of loss of MPC on mitochondrial function, we used primary (passages 1–5), or spontaneously immortalized mouse embryonic fibroblasts (MEFs) derived from *MPC1*^*+/+*^ and *MPC1*^*gt/gt*^ embryos. As found above in whole embryos, cultured *MPC1*^*gt/gt*^ primary MEFs had very low levels of MPC1 mRNA compared to *MPC1*^*+/+*^ MEFs, whereas MPC2 mRNA levels were unchanged (**Fig 2A**).

Under basal conditions, permeabilized primary *MPC1*^*gt/gt*^ MEFs showed no detectable defect in oxygen consumption rate (OCR) when pyruvate was provided as a respiratory substrate, but a major drop in OCR was observed when maximal respiration was evoked by the addition of fCCP (**Fig 2B and 2C**). This defect was abolished by supplementing the medium with methyl pyruvate (**Fig 2C**) which diffuses freely across membranes and thus bypasses the requirement for the MPC [21]. Furthermore, the MPC inhibitor UK5099 decreased pyruvate-driven OCR in *MPC1*^*+/+*^ MEFs but had no effect on *MPC1*^*gt/gt*^ MEFs (**Fig 2D**), also indicating that MPC activity was severely affected in *MPC1*^*gt/gt*^ MEFs. The presence of dichloroacetate in the experiments on permeabilized MEFs prevents inhibition of PDH by PDH kinase [22], minimizing the possibility of a bottleneck in respiration through reduced activity of PDH.

Western blotting experiments using spontaneously immortalized MEFs showed that neither MPC1 nor MPC2 could be detected in the *MPC1*^*gt/gt*^ derived cells (**Fig 2E**), suggesting that in the absence of MPC1, MPC2 is unstable and is degraded, as previously proposed by others [10,17,19,23]. Restoration of *MPC1* gene expression in *MPC1*^*gt/gt*^ MEFs by transduction with lentiviral particles containing a MPC1-Flag fusion construct led to reexpression of both MPC1 and MPC2 proteins (**Fig 2E**) and a concomitant increase in pyruvate-driven OCR compared to the parental *MPC1*^*gt/gt*^ MEFs (**Fig 2F and 2G**). Taken together, these results indicate that disruption of MPC1 expression using the gene trap strategy described above significantly diminished pyruvate entry into the mitochondria and thus the ability to utilize pyruvate as a respiratory substrate.

### MPC deficiency results in oxidative TCA cycle blockade and a compensatory increase in glutaminolysis-driven reductive TCA cycle

**Fig 3:**
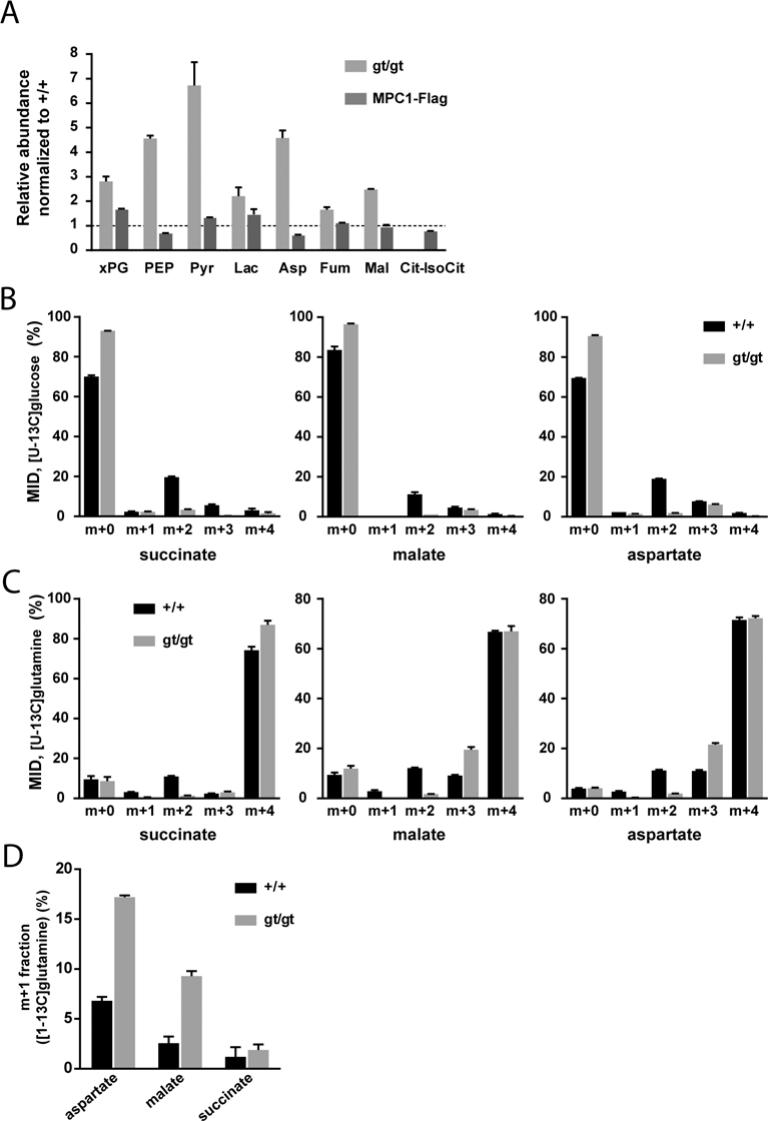
MPC deficiency alters the flux of glycolytic pyruvate into the TCA cycle and is compensated by increased glutamine-driven anaplerosis and reductive TCA cycle. **(A)** The steady-state abundance relative to *MPC1*^*+/+*^ (dotted line) of the indicated metabolites in *MPC1*^*gt/gt*^ (light grey bars) and MPC1-Flag rescued MEFs (dark grey bars) was determined by targeted metabolomics. **(B,C)** Mass isotopomer distribution (MID) of succinate, malate and aspartate in *MPC1*^*+/+*^ (black bars) and *MPC1*^*gt/gt*^ (grey bars) MEFs cultured in the presence of **(B)** [U-^13^C]glucose or **(C)** [U-^13^C]glutamine. **(D)** m+1 fraction of aspartate, malate and succinate in *MPC1*^*+/+*^ (black bars) and *MPC1*^*gt/gt*^ (grey bars) MEFs cultured in the presence of [1-^13^C]glutamine. xPG: 2-/3-phosphoglycerate; PEP: phosphoenolpyruvate; Pyr: pyruvate; Lac: lactate; Asp: aspartate; Fum: fumarate; Mal: malate; Cit-IsoCit: citrate-isocitrate.

We further assessed the metabolic consequences of MPC deletion in MEFs by metabolomics. Targeted tandem mass spectrometry was used to profile glycolytic and TCA cycle intermediates in the *MPC1*^*+/+*^, *MPC1*^*gt/gt*^ and MPC1-Flag rescued MEFs (**S1 Table**). Abolishing MPC expression caused intracellular accumulation of pyruvate, lactate and the glycolytic precursors phosphoenolpyruvate and 2/3-phosphoglycerate, as well as almost complete depletion of detectable citrate (**Fig 3A**). This pattern is indicative of defective pyruvate uptake into mitochondria and a subsequent decrease in the pyruvate-driven oxidative TCA cycle, fully consistent with our results on pyruvate-driven OCR (**Fig 2**). Furthermore, a four-fold increase in aspartate and a somewhat lesser increase in malate and fumarate were observed, also indicating reduced citrate synthase (CS) flux and accumulation of glutamine-derived molecules. In MPC1-Flag rescued MEFs, the levels of all metabolites were restored close to those observed in *MPC1*^*+/+*^ MEFs (**Fig 3A**).

In order to identify the compensatory mechanisms that are established in response to MPC deletion, we performed ^13^C-tracer experiments (for raw values, see **S2 Table**). In the presence of [U-^13^C]glucose, *MPC1*^*gt/gt*^ cells showed a substantial decrease in the M+2 mass isotopomer fraction of TCA cycle intermediates and a corresponding increase in the unlabeled fraction (**Fig 3B**). The M+2 mass isotopomer results from the incorporation of glycolytic pyruvate into citrate via MPC, PDH, and CS (**S1A Fig**). The very low M+2 fraction measured in *MPC1*^*gt/gt*^ cells indicates that this route is blocked in the absence of MPC1. Using [U-^13^C]glutamine, we found that this amino acid was the main carbon source for dicarboxylic acids in the TCA cycle (**Fig 3C**) in both *MPC1*^*+/+*^ and *MPC1*^*gt/gt*^ MEFs. The only appreciable difference between the two cell types was a decrease in the M+2 fraction and an increase in the M+3 fraction in *MPC1*^*gt/gt*^ compared to *MPC1*^*+/+*^ cells. The lack of M+2 mass isotopomers is consistent with a lack of CS activity which in the mutant, prevents synthesis of [^13^C_4_]citrate from [U-^13^C]oxaloacetate (**S1B Fig**). The higher M+3 fractions indicate an increase in reductive glutamine metabolism (**S1B Fig**). This shift was confirmed in a labeling experiment with [1-^13^C]glutamine (**Fig 3D**), which allowed us to distinguish between reductive and oxidative TCA-cycle activity (**S1C Fig**). Overall, these data demonstrate that the contribution of glucose-derived pyruvate to TCA cycle intermediates is abolished upon *MPC1* disruption, and that one compensatory mechanism established in the absence of the MPC involves glutamine-driven anaplerosis and an increase in reductive TCA cycle metabolism.

### Embryonic lethality of *MPC1*^*gt/gt*^ mice is rescued by a ketogenic diet

**Fig 4:**
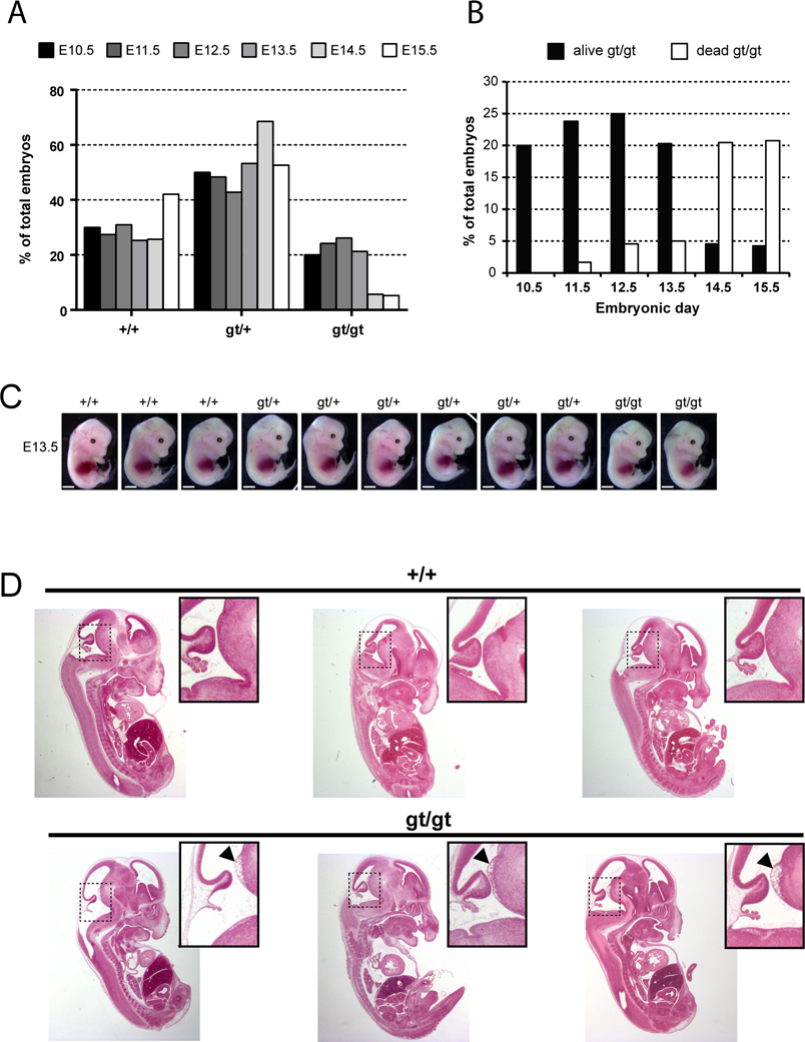
*MPC1* gene disruption results in embryonic lethality. **(A)** Genotypes frequencies of embryos derived from heterozygous *MPC1*^*gt/+*^ timed breeding (from E10.5 to E15.5). **(B)** Proportion of live and dead *MPC1*^*gt/gt*^ embryos (relative to total embryos) from E10.5 to E15.5. **(C)** Morphology of *MPC1*^*gt/gt*^ E13.5 embryos is normal compared to *MPC1*^*+/+*^ and *MPC1*^*gt/+*^ littermates. Scale bar: 1mm. **(D)** Hematoxylin and eosin (H&E) staining of paraffin-embedded E13.5 embryo sections. Insets reveal lesions (arrowheads) in the pons region of the brain stem in *MPC1*^*gt/gt*^ embryos.

The absence of *MPC1*^*gt/gt*^ offspring from breeding heterozygous *MPC1*^*gt/+*^ mice indicated embryonic lethality (**Fig 1B**). To confirm this, we conducted timed pregnancies and observed a marked drop in viability of *MPC1*^*gt/gt*^ embryos between embryonic days E12 and E14 (**Fig 4A and 4B**). Visual inspection of E13.5 *MPC1*^*gt/gt*^ embryos did not reveal any striking morphological defects compared to their wild-type and heterozygous littermates (**Fig 4C**), and histological analysis also showed that the overall internal morphology was quite normal, although specific lesions in the pons region of the embryonic brain stem were observed (**Fig 4D**). We analysed this lesion in more detail by immunostaining for cleaved (i.e. activated) Caspase-3 and no increase in the number of apoptotic cells was observed (**S4A and S4B Fig**). However, higher magnification images of the H&E stained paraffin sections showed a clear loss of tissue integrity, with numerous cellular bodies and processes detached from the rest of the tissue reminiscent of an ‘oedema-like’ morphology (**S5 Fig**). Postmitotic neurons and proliferating cells were stained respectively with tubulin beta 3 antibody (TuJ1) and Ki67 directed antibodies and no obvious changes in the numbers of these cell types was observed. A close examination of the lesion site in *MPC1*^*gt/gt*^ embryos indicates that there is a major disorganization of the periventricular zone, a region rich in proliferating cells (**S4C Fig**), although the causes underlying the morphological disruption of this region remain unclear. At this point, we are unable to say whether these lesions are responsible for the death of *MPC1*^*gt/gt*^ embryo or whether they are simply a consequence of the metabolic alteration caused by the loss of MPC1.

**Fig 5:**
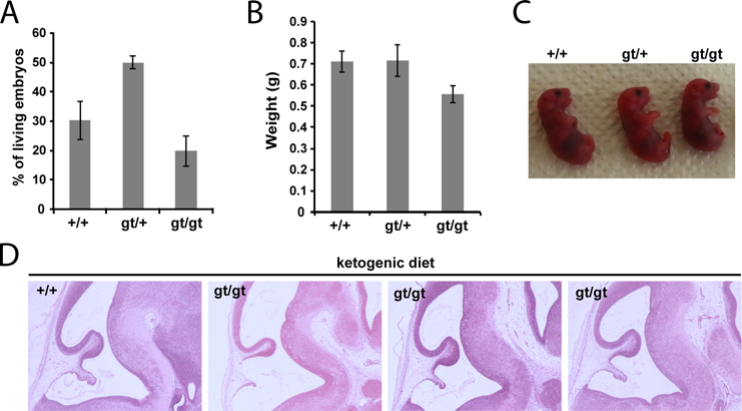
Ketogenic diet rescues embryonic lethality of *MPC1* disruption. **(A)** Genotypes of live, C-section-delivered E18.5 pups derived from heterozygous *MPC1*^*gt/+*^ breeding where the pregnant dam was fed a ketogenic diet from E8.5 onwards. **(B)** Weights of C-section-delivered E18.5 pups derived from the same breeding as in **(A)**. **(C)** Apart from a slightly smaller size, C-section-delivered E18.5 *MPC1*^*gt/gt*^ pups look outwardly normal. **(D)** Paraffin-embedded E13.5 embryo sections derived from a ketogenic diet-fed dam were stained with H&E. Note that the lesions in the pons region of *MPC1*^*gt/gt*^ embryonic brains are rescued by the ketogenic diet.

Similar to the metabolic perturbations caused by PDH deficiency [24,25], loss of MPC leads to lactic acidosis (**Fig 3A**) which is known to cause brain lesions [26] and might explain the death of the *MPC1*^*gt/gt*^ embryos. Alternatively, it is possible that some embryonic tissues may be unable to compensate for MPC disruption and are strictly dependent on pyruvate-derived acetyl-CoA for normal development. Lactic acidosis resulting from PDH deficiency can be treated with a ketogenic diet, which decreases lactate overflow and provides acetyl-CoA directly into mitochondria independently of PDH and MPC [26,27]. To test whether such a diet could also protect *MPC1*^*gt/gt*^ embryos, we maintained the pregnant dams on a ketogenic diet from E8.5 onwards. Since the ketogenic diet led to cannibalism of the pups by the mother, delivery was performed by Caesarean section at E18.5 just prior to the time of natural birth. Strikingly, the ketogenic diet resulted in the survival of the *MPC1*^*gt/gt*^ embryos up until the end of gestation (i.e. until E18.5) (**Fig 5A**), and although the weight of *MPC1*^*gt/gt*^ at E18.5 was slightly reduced compared to wild type and heterozygous littermates (**Fig 5B**), they appeared normal (**Fig 5C**). Furthermore, the lesions in the pons region of the brain stem observed in E13.5 *MPC1*^*gt/gt*^ embryos, were also prevented by the ketogenic diet (**Fig 5D**). Strikingly however, the *MPC1*^*gt/gt*^ embryos failed to survive for more than a few minutes after delivery. Nevertheless, these experiments show that the lethality seen in E13.5 *MPC1*^*gt/gt*^ embryos can be circumvented by a ketogenic diet, allowing these embryos to develop almost normally until the end of gestation.

### MPC deficiency alters the metabolic and neurotransmitter balance in the embryonic brain

**Fig 6:**
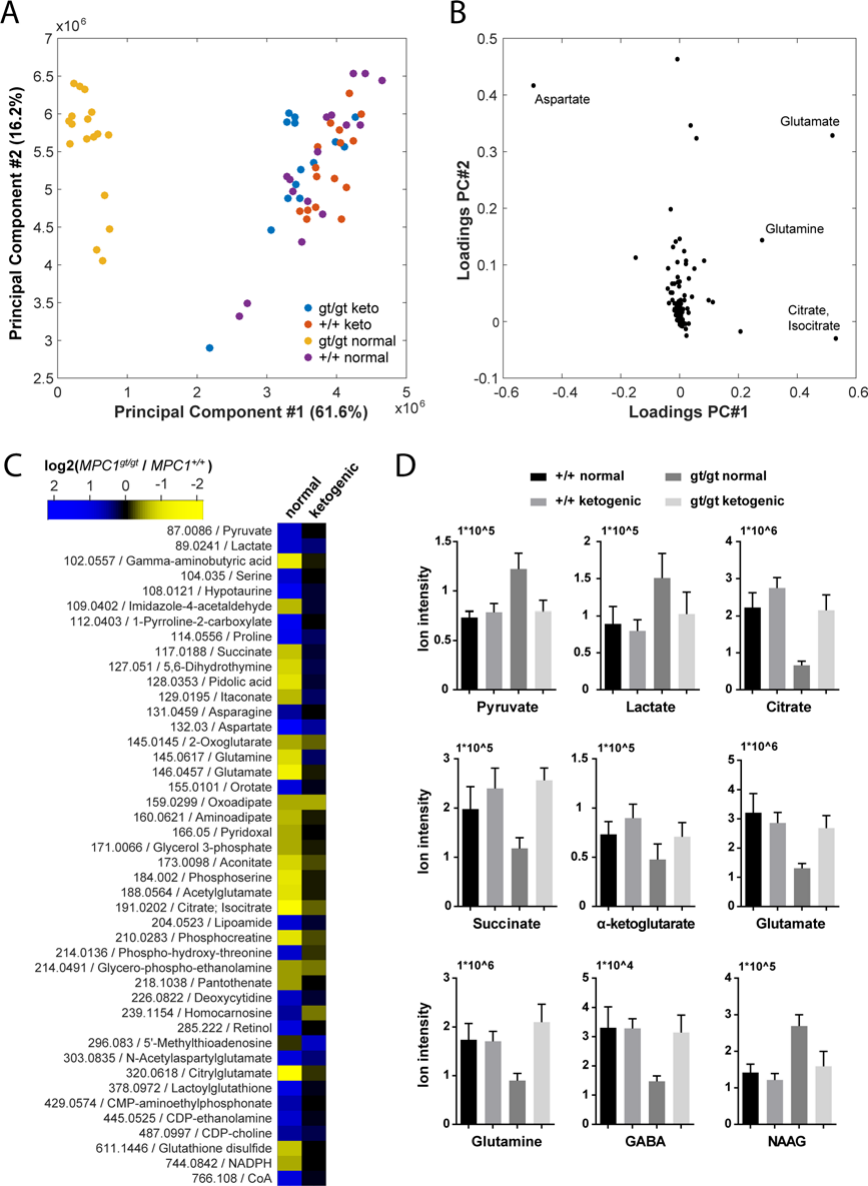
Pleiotropic alterations in the *MPC1*^*gt/gt*^ E13.5 brain metabolome are rescued by a ketogenic diet. **(A)** Principal component analysis of brain metabolome data from E13.5 *MPC1*^*+/+*^ and *MPC1*^*gt/gt*^ embryos from females maintained on normal or ketogenic (keto) diet and **(B)** underlying annotated metabolites showing highest variance among principal components. **(C)** Heat map showing fold changes of annotated metabolites (m/z is indicated) that were found to be significantly different (q-value < 0.01, abs(log2(fold change)) > 0.5) between *MPC1*^*gt/gt*^ and *MPC1*^*+/+*^ brain samples from E13.5 embryos maintained on normal or ketogenic diets. **(D)** The abundance of the indicated metabolites in the brains of E13.5 *MPC1*^*+/+*^ or *MPC1*^*gt/gt*^ embryos under normal or ketogenic diet was determined by non-targeted metabolomics.

In an attempt to understand the metabolic changes that could explain the survival of *MPC1*^*gt/gt*^ embryos until the end of gestation, we performed non-targeted metabolomic analyses on telencenphalic brain extracts from E13.5 embryos maintained on a normal or a ketogenic diet. Upon processing and annotation, a total of 222 metabolite ions could be detected (**S3 Table**). A principal component analysis of these data revealed that the most prominent metabolic changes were specific to the *MPC1*^*gt/gt*^ brain samples from animals maintained on normal diet, while *MPC1*^*gt/gt*^ ketogenic diet samples clustered with the *MPC1*^*+/+*^ samples (**Fig 6A**). Moreover, the predominant changes were all indicative of abnormal TCA cycle activity (**Fig 6B**).

To avoid possible bias due to differences in abundance and ionization efficiency, we performed a univariate analysis to systematically find all metabolites which varied between E13.5 *MPC1*^*gt/gt*^ and E13.5 *MPC1*^*+/+*^ embryos exposed to either normal or ketogenic diets. Of the 222 metabolite ions detected, 46 were found to satisfy our criteria (q-value < 0.01, abs(log2(fold change)) > 0.5) in at least one of the two dietary regimes (**Fig 6C and S2A and S2B Fig)**. The differences found in our analysis using MEFs (pyruvate, lactate, and aspartate, and TCA cycle intermediates) (**Fig 3A**) were confirmed in brain samples from animals kept on normal diet. In addition, in brain samples we found several additional changes in other pathways (e.g. proline, pyrimidine, glutathione), indicating the existence of pleiotropic effects of loss of MPC1 on the metabolic state in E13.5 *MPC1*^*gt/g*t^ brains. Interestingly, neurotransmitter levels were substantially affected in *MPC1*^*gt/gt*^ brain samples, where a decrease in glutamine, glutamate and gamma-aminobutyric acid (GABA) and an increase in N-acetylaspartylglutamate (NAAG) were observed (**Fig 6C and 6D**) suggesting that impaired pyruvate metabolism also affects the balance of neurotransmitter levels during development In addition, the phosphocreatine to creatine ratio (PCr/Cr) was low in *MPC1*^*gt/gt*^ brains following a normal dietary regime, indicative of an energy deficit *in vivo* (**S3 Fig**). Virtually all of these effects were abolished in E13.5 *MPC1*^*gt/gt*^ mice maintained on a ketogenic diet (**Fig 6C and 6D and S2B Fig**). Under these conditions, the levels of most metabolites including amino acids, GABA, and NAAG were similar to those seen in *MPC1*^*+/+*^ embryonic brains samples. Levels of lactate and aspartate were still increased in *MPC1*^*gt/gt*^ embryos under the ketogenic diet (q < 0.05) although they were only increased by 30% compared to WT embryos (**Fig 6C and 6D and S2B Fig**).

## Discussion

The aim of this study was to understand better the physiological role of the MPC in regulating metabolic homeostasis in vertebrates. In the genetic mouse model described here, we used a gene trap strategy to abolish MPC1 expression in all tissues. Homozygous disruption of *MPC1* resulted in embryonic lethality between E12 and E14 (**Fig 4A and 4B**), and this is consistent with the results on the *MPC2* knock-out mice reported by Vigueira et al. [17] who observed embryonic lethality around E11. In the latter case, a frameshift mutation was introduced two nucleotides after the initiator codon likely resulting in complete loss of the MPC2 protein. In our model, approximately 5% of mature transcripts were still present in homozygous *MPC1*^*gt/gt*^ embryos (**Fig 1D**), presumably because the splicing machinery may bypass at low frequency the splice acceptor site present in the gene trap. Even though no MPC1 protein could be detected by Western blotting (**Fig 1E**), we cannot fully exclude that low levels of functional MPC may persist. Thus, differences in the gene inactivation strategies could explain the slightly earlier death of the *MPC2* knock-out embryos as compared to the *MPC1*^*gt/gt*^ embryos.

Targeted metabolomics studies using *MPC1*^*gt/gt*^ MEFs, derived from *MPC1*^*gt/gt*^ embryos at E13.5, showed that citrate was barely detectable (**Fig 3A**), and our labeling experiments using ^13^C-tracers (**Fig 3B and 3C**) confirmed that the citrate synthase step of the TCA cycle is strongly inhibited in these cells. On the other hand, MPC disruption led to a compensatory increase in the use of glutamine as a substrate for reductive TCA cycle metabolism. In addition, our findings based on metabolic flux analysis in MEFs cultured with [U-^13^C]glucose showed that the contribution of glucose-derived pyruvate to TCA cycle intermediates is strongly inhibited in *MPC1*^*gt/gt*^ cells (**Fig 3A and 3B**) to a greater extent than previously reported [12,13]. The absence of any significant residual contribution in the present study could be explained by a more complete inhibition of the MPC mediated by insertion of the gene trap cassette, compared to the approaches based on pharmacological inhibition and RNA interference used in the previous reports. This is consistent with the fact that neither MPC1 nor MPC2 could be detected by Western blotting in *MPC1*^*gt/gt*^ MEFs (**Fig 2E**) and that no residual pyruvate import was detected making unlikely the existence of MPC-independent pyruvate import activities in these cells (**Fig 2D**). This also indicates that in our cellular model, glucose-derived pyruvate does not enter mitochondria through alternative pathways, such as pyruvate-alanine cycling as recently proposed by McCommis et al. [19], or through the action of malic enzymes that could convert pyruvate to malate in the cytosol, and convert it back to pyruvate after import of malate into the mitochondrial matrix [28]. However, we cannot exclude that these alternative routes or that additional pyruvate carriers may function in specific organs during embryogenesis, which could explain how embryos lacking the MPC can reach E12 to E14 stage. These alternative mechanisms leading to pyruvate synthesis within mitochondria may also explain why the phenotype of MPC-deficient embryos appears to be slightly less severe than the phenotype of PDH-deficient embryos which die at around stage E9 to E11 [29], and in which the contribution of pyruvate to the TCA cycle is fully inhibited.

The cause of the death of the *MPC1*^*gt/gt*^ embryos has yet to be completely elucidated although we hypothesize that metabolic acidosis is at least in part responsible for the phenotype we observe. Several years ago, Brivet et al. [30] described details of a patient showing impaired mitochondrial pyruvate import which was linked to a mutation in a gene which, *a posteriori*, was found to be *MPC1* [10]. This patient was the first child of healthy consanguineous parents and presented at birth with hypotonia, mild facial dysmorphism, periventricular cysts, marked metabolic acidosis and severe hyperlactacidemia [30]. Consistent with this study, we found increased lactic acid in the telencephalic brain of E13.5 *MPC1*^*gt/gt*^ embryos (**Fig 6C and 6D**) while Vigueira et al. [17] reported increased lactate in the blood of the *MPC2* hypomorphic mutant. Any impairment in pyruvate oxidation, whether it be due to a deficit in mitochondrial pyruvate import as in our study, decreased PDH activity [29] or defects in the TCA cycle or the respiratory chain [31], would be expected to result in increased reduction of pyruvate to lactic acid by the LDH, greater release of lactic acid into the extracellular medium and consequently to metabolic acidosis. Long term metabolic acidosis results in multiple organ failure, in particular to defects in heart contractility leading to cardiac arrest [32]. The brain is also particularly at risk during periods of metabolic acidosis and, for example, the dysfunction of neurons which accompanies inhibition of the neuronal PDH activity has previously been shown to be the cause of embryonic lethality [33]. All these results argue in favor of acidosis being a major, if not the principal cause, of embryonic lethality of MPC deficient embryos. This hypothesis is further supported by the findings that a ketogenic diet provided to the pregnant female from E8.5 onwards rescued the embryonic lethality of *MPC1*^*gt/gt*^ embryos and prevented lesions in the mesencephalon (**Fig 5**).

A ketogenic diet is commonly used to treat the lactic acidosis resulting from PDH deficiency in humans [26,27], and has been shown to have similar effects in experiments with zebrafish embryos [34]. Used therapeutically, the ketogenic diet reduces lactic acidosis probably by decreasing glucose uptake and aerobic glycolysis, the main pathway induced in mammalian cells to compensate for a deficiency in OXPHOS. The beneficial effects of the ketogenic diet may be immediate, through fueling the TCA cycle with acetyl-CoA, or delayed, through an epigenetic regulation of gene expression [35] in the embryos. In addition, the beneficial effects of the ketogenic diet may also be mediated through changes in the maternal metabolism thus changing the supply of metabolites and/or growth factors to the embryo. In our experiments, maintaining the pregnant dams on a ketogenic diet from E8.5 onwards reduced lactate accumulation allowing the *MPC1*^*gt/gt*^ embryos to complete normal gestation (**Fig 5**). We suggest that this is because the diet is able to sustain efficient oxidative metabolism, which is required during the later stages of embryogenesis for cell and tissue differentiation [1,3,4]. In agreement with this is the fact that the ketogenic diet rescued the energy deficit observed *in vivo* in the brains of E13.5 *MPC1*^*gt/gt*^ embryos **(S3 Fig)**. Moreover, in addition to the effects on lactic acid and energy balance, we observed that the ketogenic diet also normalized other metabolic parameters in the brain, including glutaminolysis which seemed abnormally elevated in untreated *MPC1*^*gt/gt*^ embryos as evidenced by reduced glutamine and glutamate levels (**Fig 6C and 6D**). Under the ketogenic diet, glutamine, glutamate, and GABA levels were increased compared to untreated *MPC1*^*gt/gt*^ embryos (**Fig 6C and 6D**) whereas the level of NAAG was decreased (**Fig 6C**). It was recently shown that GABAergic transmission in neonatal mice is essential for cortical neuron development and the establishment of a proper balance between excitation and inhibition in the adult cortex [36]. Together our results allow us to hypothesize that during embryogenesis, MPC activity is required not only for adapting energy metabolism to the needs of the developing embryo, but also in maintaining a balanced pool of major neurotransmitters and ensuring normal brain development. It is already established that PDH deficiency is associated with severe neurological phenotypes such as developmental defects, ataxia, cognitive delay and epilepsy [24–27], the latter being caused by impaired energetic status and abnormal neurotransmitter metabolism [26]. In future experiments, it will be of interest to evaluate further the role of the MPC in modulating neurotransmitter levels and in regulating brain function.

Despite the ability of the ketogenic diet to restore normal metabolism and gestation of the *MPC1*^*gt/gt*^ embryos (**Figs 5 and 6 and S2 Fig**), the newborn pups survive for only a few minutes post-delivery. This suggests that without nutritional support from the dam, which provides a continuous source of glucose and ketone bodies, *MPC1*^*gt/gt*^ pups were not able independently to meet their energetic needs during the post-natal starvation state. Loss of pyruvate oxidation and ketogenic supply in the *MPC1*^*gt/gt*^ pups may be further exacerbated by the fact that autophagy-driven gluconeogenesis, an important source of energy during the post-natal period [37], is probably impaired in these newborn animals. Indeed, recent reports indicate that liver-specific ablation of MPC activity diminishes the gluconeogenic flux because of the relatively low efficiency of compensatory pathways such as glutaminolysis and pyruvate/alanine cycling in liver [18,19]. Our results show that global loss of MPC activity is incompatible with embryonic development and neonatal survival in mammals.

## Material and Methods

### Ethics statement

Mice were euthanized by CO2 inhalation. All experimental procedures were performed according to guidelines provided by the Animal Welfare Act and Animal welfare ordinance, the Rectors' Conference of the Swiss Universities (CRUS) policy and the Swiss Academy of Medical Sciences / Swiss Academy of Sciences' Ethical Principles and Guidelines for Experiments on Animals, and were approved by the Geneva Cantonal Veterinary Authority (authorization number: 1027/3907/1).

### Mice

Mice bearing the *MPC1*^*gt*^ allele were generated by the Texas A/M institute for Genomic Medicine (TIGM) using the OmniBank ESC clone OST39041. Timed matings were set up at the end of the day and the presence of a vaginal plug was checked the following morning. This time point was taken as E0.5. To rescue embryonic lethality with a ketogenic diet, timed matings were set up as above and normal food was replaced at 8 dpc by a diet containing 75% fat and 10% protein (Ketogenic diet XL75:XP10, Kliba Nafag, Switzerland).

### Cell culture and lentiviral transduction

Cells were grown in Dulbecco’s modified Eagle’s medium (DMEM) containing 25mM glucose supplemented with 2mM L-Glutamine, 10% FBS and Penicillin/Streptomycin at 37˚C, 5% CO_2_.

MEFs were isolated from E13.5 embryos as described elsewhere [38]. Briefly, the embryos were dissected out, internal organs were removed and the carcasses were minced with a razor blade and incubated in 0.25% Trypsin/EDTA for 15 min at 37˚C. After addition of growth medium, cells were spun down and plated. Experiments with primary cells were carried out no more than 5 passages after isolation. Immortalized MEFs appeared spontaneously in primary MEFs cultures after continued passaging. Rescued MEFs were obtained by transduction of immortalized *MPC1*^*gt/gt*^ MEFs with lentiviral particles containing the C-terminally Flag-tagged MPC1 coding sequence under the control of the EF1-alpha promoter. Generation of this construct as well as the lentiviral transduction procedure has been described previously [39].

### RT-qPCR

RNA was extracted from whole embryo homogenates or from cultured cells using TRIzol reagent (Ambion) according to the manufacturer’s instructions. Reverse transcription was carried out using M-MLV reverse transcriptase (Invitrogen) and random primers (Promega) from 2 μg of total RNA according to the manufacturer’s instructions. Quantitative PCR using SsoFast Evagreen supermix (BioRad) was performed according to the manufacturer’s protocol. The following primers were used: MPC1 F: GACTATGTCCGGAGCAAGGA; MPC1 R: TAGCAACAGAGGGCGAAAGT; MPC2 F: TGTTGCTGCCAAAGAAATTG; MPC2 R: AGTGGACTGAGCTGTGCTGA; 28S F: TTGAAAATCCGGGGGAGAG; 28S R: ACATTGTTCCAACATGCCAG. The relative abundance of the MPC1 and MPC2 transcripts in each sample was determined by normalizing to 28S rRNA using BioRad CFX manager software.

### Western blot

Total cell lysates were prepared by lysing cells in RIPA lysis buffer for 15 min on ice and removal of insoluble material by centrifugation at 16000g for 10 min at 4˚C. Protein content was measured using the Bio-Rad protein assay and equal protein amounts in 1x Laemmli buffer were used for SDS-PAGE. For immunoblotting, proteins were transferred electrophoretically to nitrocellulose membranes and exposed to the following primary antibodies: anti-MPC1 (HPA045119, Sigma), anti-MPC2 (home-made), anti-TOM20 (sc-11415, Santa-Cruz), anti β-tubulin (T4026, Sigma). Images of Western blotting were uniformly treated for contrast enhancement using Adobe Photoshop.

### Oxygen consumption

Measurement of oxygen consumption was performed using a Seahorse XF^e^24 Flux Analyzer (Seahorse Biosciences). 30’000 cells were seeded in XF24 cell culture microplates and grown overnight in DMEM containing 10% FBS, 2mM L-Glutamine, 25mM Glucose, and Penicillin/Streptomycin. Experiments on primary or immortalized, permeabilized MEFs were carried out at 37°C in Mitochondrial Assay Solution (MAS, containing 70 mM sucrose, 220 mM mannitol, 10 mM KH_2_PO_4_, 5 mM MgCl_2_, 2 mM Hepes, 1 mM EGTA, 0.2% fatty acid free BSA; pH 7.2). Cells were permeabilized with 1 nM XF Plasma Membrane Permeabilizer reagent (Seahorse Bioscience) and provided with 5 mM pyruvate, 0.5 mM malate, 2 mM dichloroacetate and 1 μM oligomycin one hour before the assay. Basal oxygen consumption was measured before injection. At the times indicated, the following compounds were injected: fCCP (final concentration 0.4 μM (primary MEFs) or 2 μM (immortalized MEFs)), succinate/rotenone (10 mM/1 μM), antimycin A (1 μM). Each measurement loop consisted in 30 sec mixing, 1 min waiting, and 2 min measuring oxygen consumption. For immortalized cells, OCR data were corrected for cell number by nuclear staining with DAPI.

### Histology and immunofluorescence

Embryos were surgically removed at the times indicated and immediately fixed in Bouin’s fixative solution, dehydrated, paraffin-embedded, and sectioned at 4 μm. Sections were mounted on glass slides and stained with haematoxylin and eosin. For cryosections, embryos were fixed for 1 hr at room temperature in 4% paraformaldehyde, rinsed twice in PBS and cryoprotected in 15% sucrose/PBS for at least 48 hrs. After inclusion in 7.5% gelatin/15% sucrose/PBS, blocks containing the embryos were snap-frozen in liquid nitrogen-cold isopenthane before sectioning in a cryostat. Sections were collected on Superfrost Plus glass slides (ThermoScientific), allowed to dry for ~30 min and stored at -20°C before immunostaining. For immunofluorescence studies, cryosections were rehydrated in PBS, and the excess embedding matrix was removed by 1 min incubation in 37°C pre-warmed PBS. Sections were permeabilized in 0.1% Triton X-100/PBS for 20 min, rinsed 3 times in PBS, and incubated for 30 min in blocking buffer containing 3% bovine serum albumin/0.1% Tween 20 in PBS. Incubations with primary antibodies were performed for 3 hrs at room temperature, before rinsing 3 times in PBS and incubation for 1 hr at room temperature with secondary antibodies. Nuclei were stained with DAPI, sections were mounted in FluorSave (Millipore) and observed using a Cytation 3 Cell Imaging apparatus. Antibodies used were Cleaved Caspase-3 (Asp175)(#9664, Cell Signaling), tubulin β 3 (TuJ1 clone, 801201, BioLegend) and Ki67 (ab15580, Abcam).

### Metabolomics and ^13^C labeling analysis of MEFs

For labeling experiments, the tracer medium was obtained by replacing the carbon substrate of interest with ^13^C labeled glucose or glutamine (Cambridge Isotopes). Both, metabolomics and labeling experiments were performed as follows: 6–8 x 10^4^ cells/well were seeded in a 6-well plate (Nunc) and allowed to attach for approximately 12 h in the presence of unlabeled DMEM. The medium was then completely removed, and cells were washed with 1x PBS. Fresh medium, labeled or unlabeled as appropriate, was added and cells were incubated for further 24 h. Three replicates per condition were performed. For sampling, the medium was removed and cells were washed twice with 75 mM ammonium carbonate buffer (pH 7.4) and quenched by snap freezing the plate in liquid N_2_. Plates were stored at -80°C until proceeding with metabolite extraction.

Metabolite extraction was performed by addition of 1.8 mL of cold (-20^°^C) extraction solution (acetonitrile/methanol/water (2:2:1)) to each well. For targeted metabolomics experiments, 200 μL of a uniformly ^13^C labeled *E*. *coli* metabolite extract was added as internal standard [40]. For pyruvate measurements, the extraction solution above also contained 25 μM phenylhydrazine for derivatization, and 100 μL of a 5 μM [U-^13^C]pyruvate solution as an internal standard [41]. After 1 h incubation at -20^°^C, the bottom of each well was scraped and the extract was collected in a 2 mL microcentrifuge tube. Extracts were centrifuged (4°C, 10,000 rpm, 10 min) to remove cell debris, and the supernatants were transferred to fresh tubes. For targeted metabolomics, supernatants were evaporated to complete dryness, while the pellets containing the cell debris were used to normalize metabolite concentrations to cellular protein. Pellets were incubated with CellLytic lysis reagent (Sigma) and protein content was quantified using the Bradford assay.

### Targeted metabolomics

Dried samples were resuspended in 100 μL deionized water, and 10 μL aliquots were injected into a Waters Acquity UPLC (Waters Corporation, Milford, MA) with a Waters Acquity T3 column coupled to a Thermo TSQ Quantum Ultra triple quadrupole instrument (Thermo Fisher Scientific) with negative-mode electrospray ionization. Compound separation was achieved by a gradient of two mobile phases (A) 10 mM tributylamine, 15 mM acetic acid and 5% (v/v) methanol, and (B) 2-propanol [41,42]. Acquisition of mass isotopomer distributions of intact and fragmented carbon backbones was done as previously described [43]. Peak integration was performed using an in-house software. Metabolites were quantified by normalizing the peak area of each compound to the respective signal from the internal standard, additionally compared to the calibration curve with known metabolite concentrations. Fractional labeling and MIDs were calculated as previously described [44], and corrected for naturally occurring ^13^C [45].

### Non-targeted metabolomics analysis of brain samples

The telencephalic brain was dissected from E13.5 embryos harvested from dams fed either a normal or a ketogenic diet. Metabolites were then extracted from the ca. 10 mg brain samples. The brain pieces were homogenized in 1 mL cold 70% (v/v) ethanol with a TissueLyser (Qiagen). Extraction was continued with addition of 7 mL of 70% (v/v) ethanol preheated to 75^°^C for 2 min, followed by removal of cell debris by centrifugation (4^°^C, 4,000 rpm, 15 min). Extracts were stored at -20°C until mass spectrometric analysis. Non-targeted metabolomics was performed by flow injection analysis on a 6550 Agilent QTOF instrument as described previously [46]. Briefly, profile spectra were recorded in negative ionization from m/z 50 to 1000 mode at 4 GHz high-resolution mode. Ion annotation was based on accurate masses using a tolerance of 0.001 a.m.u. and KEGG mmu database, accounting systematically for–H^+^ and F^-^ ions, sodium and potassium adducts, and heavy isotopes. The full annotated ion list is provided in **S3 Table**.

### Statistical analyses

Differences in mRNA expression levels were assessed by two-way ANOVA using the GraphPad Prism software. Statistical analysis of metabolomics data was performed by using Matlab R2015a and software developed in-house. Significance of changing metabolites between groups (*MPC1*^*+/+*^ and *MPC1*^*gt/gt*^) was calculated from Student’s t-test distribution, and p-values were adjusted to account for false discovery rate [47].

## Supporting Information

S1 Fig Schematics for carbon transitions during metabolic flux analysis in *MPC1*^*+/+*^ and *MPC1*^*gt/gt*^ rescued MEFs. Schematic of carbon transitions in the labeling experiment of TCA-cycle, adopted from Metallo et al. [48], with **(A)** [U-^13^C]glucose (blue = labeled, white = unlabeled), **(B)** [U-^13^C]glutamine (green = labeled, white = unlabeled), and **(C)** [1-^13^C]glutamine (red = labeled, white = unlabeled). ME: malic enzyme; PDH: pyruvate dehydrogenase; AcCoA: acetyl-CoA.
(PDF)

S2 Fig Maps of the metabolic changes between the E13.5 brain of *MPC1*^*+/+*^ and *MPC1*^*gt/gt*^ embryos on different diets. Metabolites are depicted by the circles of particular size representing the significance (FDR corrected q-values) and by the color, representing the fold changes (green = less, red = more) normalized to *MPC1*^*+/+*^. **(A)** normal or **(B)** ketogenic diet.
(PDF)

S3 Fig *In vivo* energy deficit in *MPC1*^*gt/gt*^ embryonic brains is rescued by a ketogenic diet. Phosphocreatine to creatine ratio in the E13.5 telencephalon from embryos of the indicated genotype and maintained on the indicated diet. Data were extracted from the non-targeted metabolomics experiment. **** p<0.0001, t-test.
(TIF)

S4 Fig Immunofluorescence staining of the brain lesion sites in *MPC1*^*+/+*^ or *MPC1*^*gt/gt*^ E13.5 embryos from dams maintained on a normal diet. Low **(A)** and high **(B,C)** magnification images of cryosections from E13.5 embryos of the indicated genotype immunostained using anti-Cleaved Caspase3 (green **(A,B)**), anti-Ki67 (green **(C)**) or TuJ1 (red **(A,B,C)**) antibodies. Nuclei were stained with DAPI. Merged green and blue **(B)** or green and red **(C)** channels are also shown. Scale bars: 200 μm **(A)** or 100 μm **(B,C)**. BF = bright field. Cb = cerebellum. Cp = choroid plexus. P = pons. Arrowheads indicate apoptotic cells.
(TIF)

S5 Fig High magnification of the pons region of the brainstem in H&E stained *MPC1*^*+/+*^ or *MPC1*^*gt/gt*^ embryos. Three different embryos of each genotype were analysed. Scale bar: 50 μm.
(TIF)

S1 Table Data for targeted metabolomics on MEFs. (XLS)

S2 Table Data for labeling experiments on MEFs. (XLS)

S3 Table Data for brain non-targeted metabolomics. (XLS)
