# Supplementary figures and images for: Embryonic Lethality of Mitochondrial Pyruvate Carrier 1 Deficient Mouse Can Be Rescued by a Ketogenic Diet
Source: PLoS Genet. 2016 May 13;12(5):e1006056. doi: 10.1371/journal.pgen.1006056 (PMC4866774; doi:10.1371/journal.pgen.1006056)

A

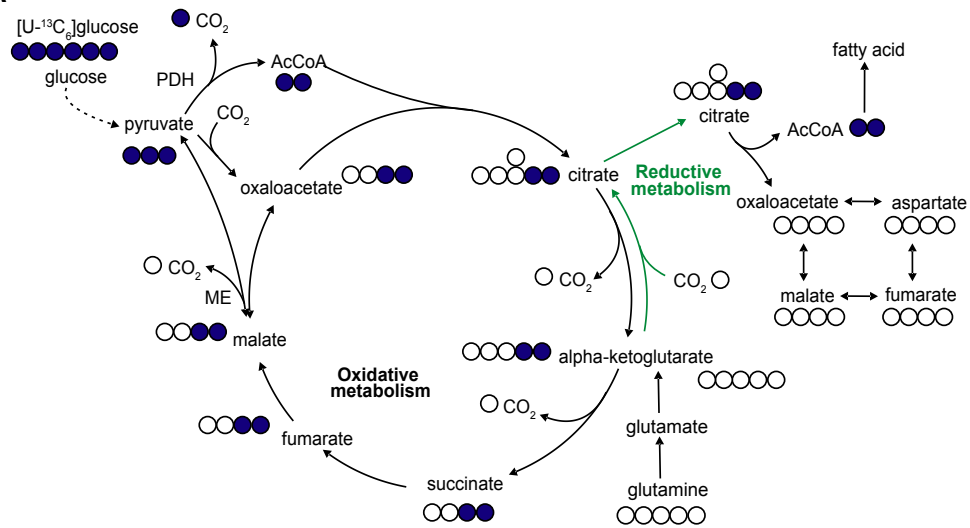

B

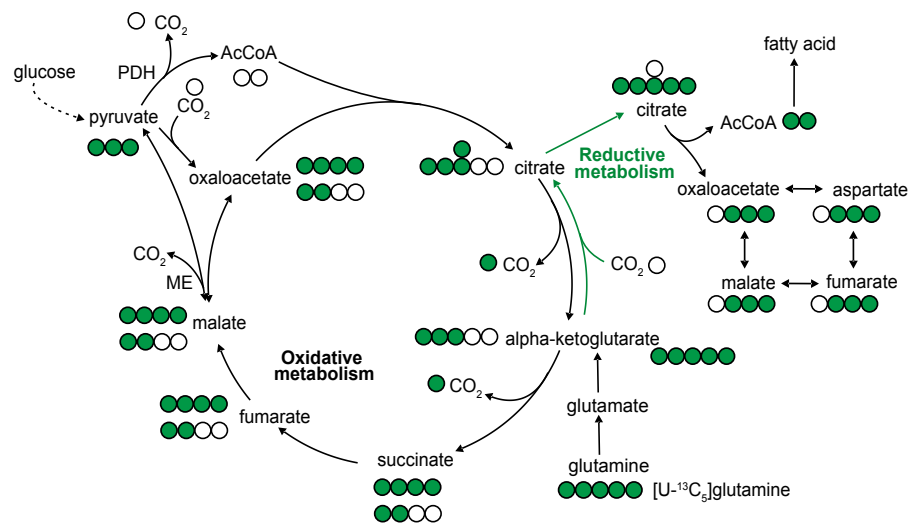

C

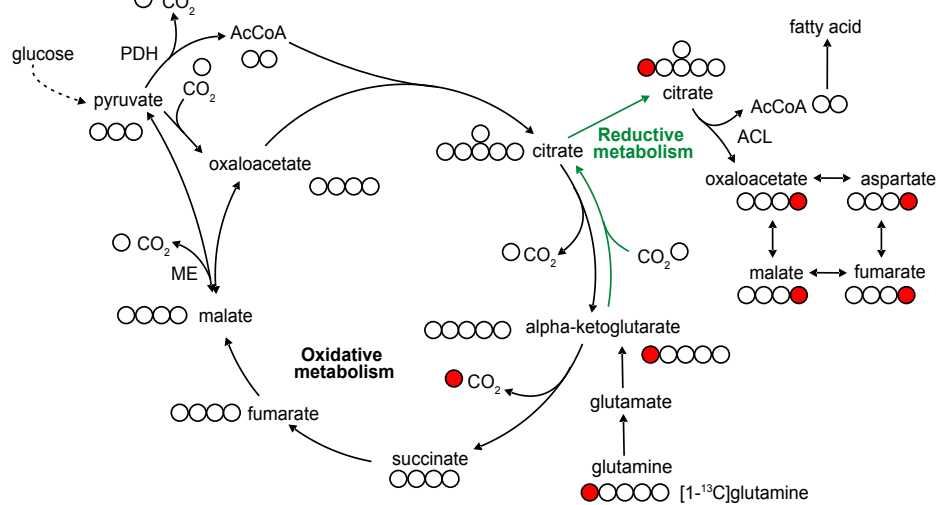

Supplement: S1 Fig — Schematic of carbon transitions in the labeling experiment of TCA-cycle, adopted from Metallo et al. [48], with (A) [U-13C]glucose (blue = labeled, white = unlabeled), (B) [U-13C]glutamine (green = labeled, white = unlabeled), and (C) [1-13C]glutamine (red = labeled, white = unlabeled). ME: malic enzyme; PDH: pyruvate dehydrogenase; AcCoA: acetyl-CoA. (PDF) [file pgen.1006056.s001.pdf]

A

normal diet:  $MPC1^{gt/gt} / MPC1^{+/+}$ 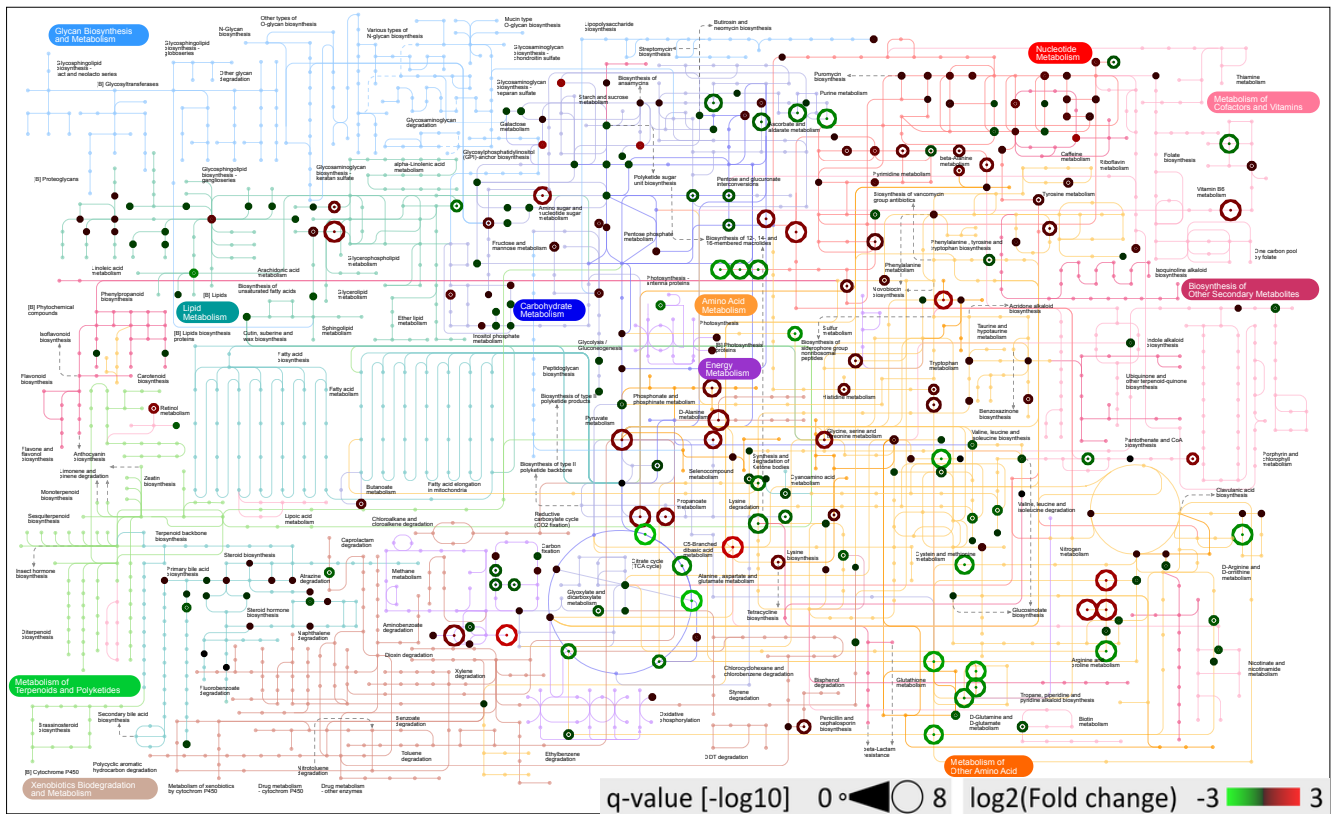

B

ketogenic diet:  $MPC1^{gt/gt} / MPC1^{+/+}$ 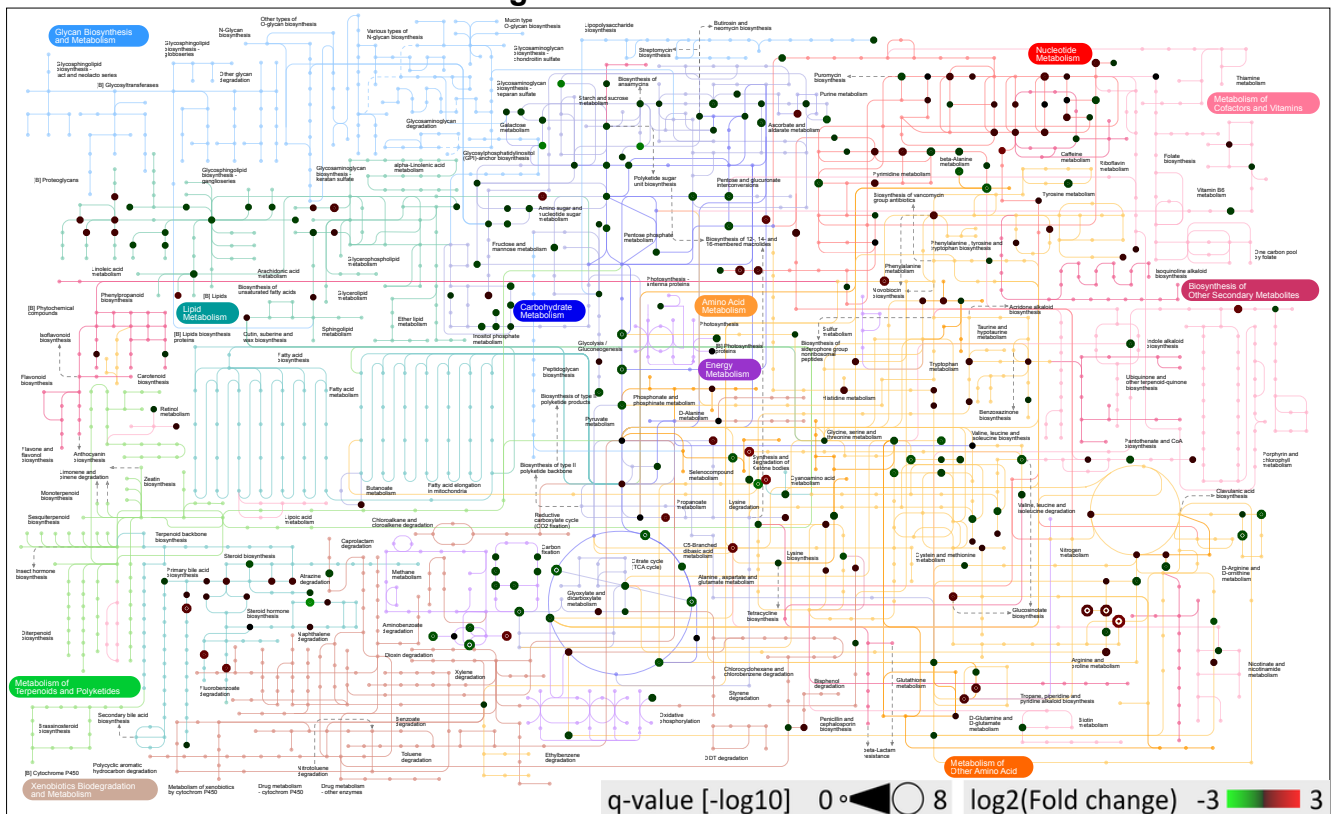

Supplement: S2 Fig — Metabolites are depicted by the circles of particular size representing the significance (FDR corrected q-values) and by the color, representing the fold changes (green = less, red = more) normalized to MPC1+/+. (A) normal or (B) ketogenic diet. (PDF) [file pgen.1006056.s002.pdf]

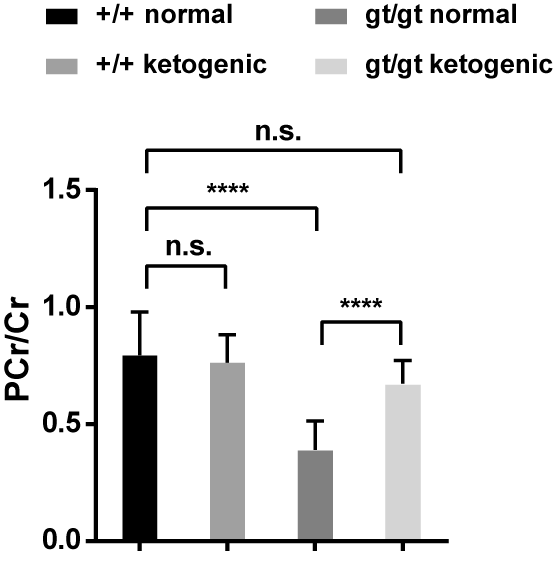

Supplement: S3 Fig — Phosphocreatine to creatine ratio in the E13.5 telencephalon from embryos of the indicated genotype and maintained on the indicated diet. Data were extracted from the non-targeted metabolomics experiment. **** p<0.0001, t-test. (TIF) [file pgen.1006056.s003.tif]

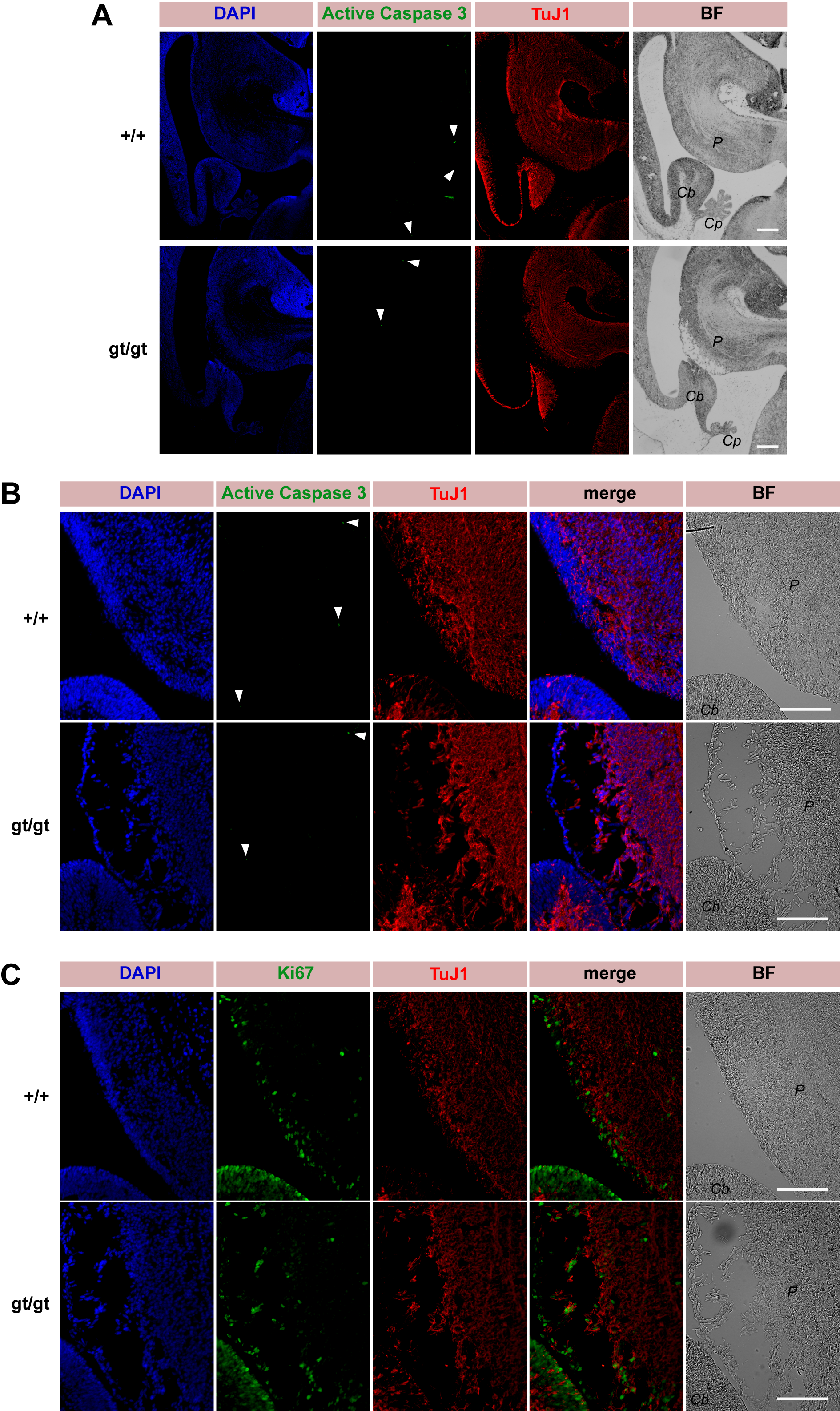

Supplement: S4 Fig — Low (A) and high (B,C) magnification images of cryosections from E13.5 embryos of the indicated genotype immunostained using anti-Cleaved Caspase3 (green (A,B)), anti-Ki67 (green (C)) or TuJ1 (red (A,B,C)) antibodies. Nuclei were stained with DAPI. Merged green and blue (B) or green and red (C) channels are also shown. Scale bars: 200 μm (A) or 100 μm (B,C). BF = bright field. Cb = cerebellum. Cp = choroid plexus. P = pons. Arrowheads indicate apoptotic cells. (TIF) [file pgen.1006056.s004.tif]

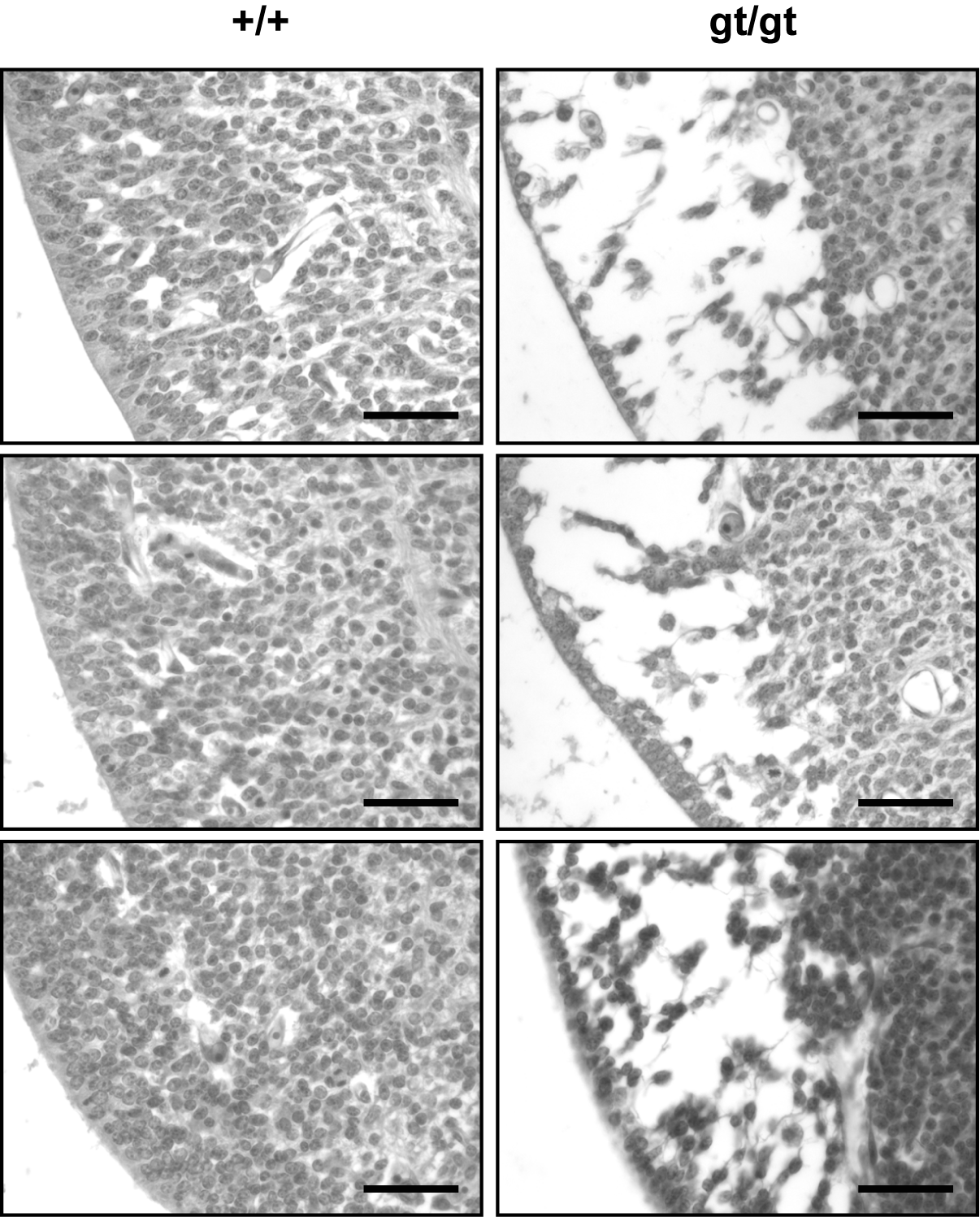

Supplement: S5 Fig — Three different embryos of each genotype were analysed. Scale bar: 50 μm. (TIF) [file pgen.1006056.s005.tif]
